# Direct and indirect effects of COVID-19 on perinatal outcomes in low- and middle-income countries

**DOI:** 10.12688/gatesopenres.13156.2

**Published:** 2021-07-21

**Authors:** Felisita Tupou Ratu, Kathleen Ryan, Netsanet Workneh Gidi, Ilisapeci Vereti, Tsinuel Girma, Jeremy Oats, Ingrid Bucens, Alexandra Robinson, Claire von Mollendorf, Fiona M. Russell

**Affiliations:** 1 Murdoch Children’s Research Institute, Melbourne, Australia; 2 College of Public Health and Medical Sciences, Jimma University, Jimma, Ethiopia; 3 Center for International Health, LMU University Hospital, Munich, Germany; 4 Colonial War Memorial Hospital, Ministry of Health and Medical Services, Suva, Fiji; 5 Melbourne School of Population and Global Health, The University of Melbourne, Melbourne, Australia; 6 National Hospital Guido Valadares, Dili, Timor-Leste; 7 UNFPA Pacific Sub Regional Office, Suva, Fiji; 8 Department of Paediatrics, The University of Melbourne, Melbourne, Australia

**Keywords:** COVID-19, perinatal outcomes, maternal outcomes, indigenous, Pacific Islands, comorbidities

## Abstract

Similar to previous outbreaks, the coronavirus disease 2019 (COVID-19) pandemic will have both direct and indirect effects on perinatal outcomes, especially in low- and middle-income countries. Limited data on the direct impact of severe acute respiratory syndrome coronavirus 2 (SARS-CoV-2) infection during pregnancy shows women who are Black, obese and with co-morbidities are at higher risk of hospitalisation due to COVID-19. Younger age groups in Africa and South Asia have shown increased COVID-19 mortality. Indigenous pregnant women in Pacific Island countries are likely to be high risk for severe outcomes from COVID-19 due to high rates of diabetes and obesity. It is important to involve pregnant women in research, especially with regards to vaccine development and therapeutics.

## Disclaimer

The views expressed in this article are those of the author(s). Publication in Gates Open Research does not imply endorsement by the Gates Foundation.

## Open letter

Improvements in maternal and newborn health are essential to attain the 2030 UN SDG health targets ^1^. The coronavirus disease 2019 (COVID-19) pandemic will have a substantial impact on perinatal outcomes in low- and middle-income countries due to: the direct effect of severe acute respiratory syndrome coronavirus 2 (SARS-CoV-2); and its indirect effects on the disruption of essential maternity and newborn services ^2^. Whitehead *et al.* stressed the importance of including pregnant women in clinical trials as SARS-CoV-2 drugs and vaccines are developed ^3^. However, it is also necessary to understand the perinatal epidemiology to determine whether inclusion into clinical trials is required.

The clinical manifestations of SARS-CoV-2 are rapidly evolving. So far, there are few data on the direct impact of SARS-CoV-2 infection during pregnancy. However, recent findings from a UK study are disturbing. This prospective cohort study from 194 hospitals found that approximately 100 pregnant women are hospitalised with COVID-19 each week. Of the 427 women included in the study, 9% needed intensive care unit (ICU) admission, four (1%) needed extra corporeal membrane oxygenation (ECMO), five (1%) mothers and five babies died ^4^. There was a strong association between admission with COVID-19 and being Black or of minority ethnicity, having a comorbidity (e.g. diabetes) and being obese/overweight ^4^. A recent US study found that compared to controls, 16 placentas from women with SARS-CoV-2 infection exhibited a higher frequency of placental injury reflecting irregularities in oxygenation associated with adverse perinatal outcomes ^5^. This corroborates with other reports of COVID-19 cases having large and small blood vessel pathology ^6^. Intrauterine vertical transmission of SARS-CoV-2 is possible, but direct evidence is lacking. There are only two reports of potential vertical transmission in three newborns ^7^.

Of key importance to low- and middle-income countries is whether SARS-CoV-2 can be transmitted during breastfeeding. In these settings, early initiation and exclusive breastfeeding until six months of age is recommended by WHO, and exclusive breastfeeding has been shown to reduce infant morbidity and mortality. Current WHO and UNICEF guidelines recommend continuation of breastfeeding in SARS-CoV-2 positive mothers with appropriate prevention strategies, such as wearing of masks and hand hygiene. There is, however, sparse evidence with small case numbers to show that SARS-CoV-2 is present in human milk and no compelling evidence regarding its role in vertical transmission ^8^.

Now that COVID-19 vaccines are available, the WHO SAGE committee has published a roadmap which outlines how to prioritise vaccination depending on vaccine availability and disease burden and risk. The roadmap acknowledges that pregnant women have been disadvantaged with regards to the development and use of vaccines in pandemics. Data needs to be generated for pregnant women and pregnant women should be included in roadmap stage 3 - when evidence should be available to determine whether the benefits of COVID-19 vaccination outweighs the risk of SARS-CoV2 infection and potential severe COVID-19 ^9^.

It is known that being Black or of South Asian ethnicity, having diabetes and obesity are key risk factors for COVID-19 mortality ^10^. Elevated blood glucose levels have not only been shown to be an independent risk factor for death in COVID-19 patients, but also a predictor of subsequent clinical deterioration ^11^. Indigenous populations are also one of the most vulnerable COVID-19 populations. Pacific Island Countries have one of the highest rates of diabetes and obesity in the world ^12, 13^. A recent perinatal review in Fiji, found that managing maternal diabetes was one of the key recommendations to improve perinatal outcomes and prevent stillbirths (J. Oats, personal communication). This situation is likely to be the same in other Pacific Island Countries. So far, Pacific Island Countries have averted community transmission of COVID-19, however, it is likely that importations will reoccur as the countries open up.

Recent data highlighted by Klugman *et al*. ^14^ shows higher mortality in younger age groups in people of colour and in poorer communities in Africa. This also has implications for young pregnant women in Pacific Island Countries.

It is known that infection with influenza virus increases the risk of maternal hospitalisation ^15^ and poor perinatal outcomes ^16^. As such, influenza vaccination is recommended for pregnant women ^17^. SARS-CoV-2 is likely to become a seasonal virus, similar to influenza. During the 2003 SARS outbreak, reports indicated that pregnant women infected with SARS had worse outcomes than non-pregnant women ^18^. Perinatal epidemiology and immunology have been omitted in the previous coronavirus epidemics (MERS and SARS-1). Pregnant women have decreased T and B cell counts ^19^ and increased expression of the angiotensin-converting enzyme 2 (ACE-2) receptor ^20^ which may increase susceptibility to SARS-CoV-2. Pregnancy is a relative immunodeficient and pro-inflammatory state raising concerns regarding the effects of SARS-CoV-2 on pregnant patients ^21^. A recent study described a pre-eclampsia-like syndrome in six COVID-19 infected pregnant women with severe pneumonia ^22^. Viral hyperstimulation in pregnant women has been shown to have adverse effects on foetal brain development ^21^. So far, nothing has been published on the immune response to SARS-CoV-2 in pregnancy.

Reports are surfacing about the impact of social distancing, cessation of transport, and pregnant women giving birth in home in low-and middle-income countries. These measures have made accessing essential health care much more difficult. In India, a 21% reduction in institutional deliveries have been reported ^23^. In addition, health staff are being diverted and some facilities are experiencing limits on equipment required for emergency obstetric care, such as blood supplies needed for post-partum haemorrhage. It is likely that all services ranging from contraceptive access to essential antenatal care will be affected without focused attention and effort. Reports in the media of unwanted pregnancies and lack of access to terminations, have come out of India, where community health workers responsible for distribution of contraception and reproductive health services have been diverted to do coronavirus screening and referrals. A mere 10% decline in contraception use in low- and middle-income countries could result in an additional 15 million unintended pregnancies over the course of a year ^24^. In the past, several African countries have suffered from the indirect effects of Ebola epidemics, resulting in the same number of maternal and newborn deaths as those caused by the direct effect of Ebola.

Early in the pandemic a study modelled the indirect effect of the pandemic on maternal and child health and resultant additional deaths in 118 LMICs using three possible scenarios ^2^. The scenarios varied in the extent of reduction in essential health intervention coverage (including a reduction in antenatal and postnatal care ranging from 18.5–51.9%) and increase in prevalence of wasting. It was estimated that under-5 child deaths could increase by 9.8–44.7% per month and maternal deaths by 8.3–38.6% per month depending on the associated disruptions ^2^. Data collected by the Population Council in five informal settlements in Nairobi in April 2020 found that women were twice as likely as men to miss essential health services. Missed services including family planning and antenatal care ^25^. In addition, midwives in Kenya, Uganda and Tanzania, reported a reduction in the number of women attending maternal health clinics, and an increase in delayed labour presentations to hospital and insufficient antenatal care ^26^.

A systematic review which included LMICs evaluated the likely impact of the COVID-19 pandemic on immunisation coverage and the factors contributing to service disruption. The review found a reduction in vaccination coverage, reduction in doses administered and an increase in polio cases in polio endemic countries ^27^.

It is important to understand the direct and indirect effects of COVID-19 on routine essential health services and perinatal outcomes of SARS-CoV-2. It is vital to invest in research, especially in low- and middle-income countries to undertake special epidemiological studies in pregnant women, as large, existing datasets are usually not available to undertake rapid analyses of clinical data as in high-income countries, nor measure a future vaccine’s impact. In 1875, one-third of the Fijian population died from a measles epidemic sweeping through a non-immune population ^28^. To avoid this, and end the current pandemic, 7 billion people need to be vaccinated, including pregnant women. Delays in including this vulnerable population in COVID-19 vaccine and other intervention studies, may erode the gains made in maternal and child health, globally, especially in resource-poor settings.

In terms of COVID-19 research in LMICs, it has been suggested that this is incorporated into public health and clinical activities to avoid these vital services being diverted, and also that this research should be driven by local investigators ^29^. The WHO has published a number of generic protocols for COVID-19 research and response, including surveillance, serology and case investigation ^30^. In 2020 the United Nations launched a global humanitarian response plan to fund the COVID-19 response in the poorest countries with both public and private sector support ^31^.

## Data availability

### Underlying data

No data are associated with this article.
