# Supplementary material for: Direct and indirect effects of COVID-19 on perinatal outcomes in low- and middle-income countries
Source: Gates Open Res. 2021 Jul 21;4:108. Originally published 2020 Jul 16. [Version 2] doi: 10.12688/gatesopenres.13156.2 (PMC11259593; doi:10.12688/gatesopenres.13156.2)
Supplement: Peer review history

# Reviewer report: Reviewer response for version 2

**DOI:** 10.21956/gatesopenres.14465.r36876

**Published:** 2024-07-19

**Authors:** Atakan Tanacan

**Affiliations:** 1 Department of Obstetrics and Gynecology, Turkish Ministry of Health Ankara City Hospital, University of Health Sciences, Ankara, Turkey

I have read the manuscript with great interest. The authors have shared their opinions about the impact of COVID-19 on perinatal outcomes in low-and-middle income countries in the light of the current literature. The authors have performed the recommended revisions and the manuscript has improved with the recommended changes. The paper may be indexed in its present form.

Does the article adequately reference differing views and opinions?

Yes

Are all factual statements correct, and are statements and arguments made adequately supported by citations?

Yes

Is the Open Letter written in accessible language?

Yes

Where applicable, are recommendations and next steps explained clearly for others to follow?

Yes

Is the rationale for the Open Letter provided in sufficient detail?

Yes

Reviewer Expertise:

perinatology, high-risk pregnancy, prenatal diagnosis

I confirm that I have read this submission and believe that I have an appropriate level of expertise to confirm that it is of an acceptable scientific standard.

# Reviewer report: Reviewer response for version 1

**DOI:** 10.21956/gatesopenres.14351.r29541

**Published:** 2020-09-28

**Authors:** Salut Muhidin

**Affiliations:** 1 Department of Management, Macquarie Business School, Macquarie University, Sydney, NSW, Australia

The discussions on direct and indirect effects of COVID-19 are very time-appropriate and important during this current situation. However, the focus should not only be limited to low and middle income countries, it could also be expanded to disadvantaged communities. 

 The main effects are getting bigger and serious due to limited access that are often experienced by low income communities, regardless in which country they live.

Does the article adequately reference differing views and opinions?

Yes

Are all factual statements correct, and are statements and arguments made adequately supported by citations?

Partly

Is the Open Letter written in accessible language?

Yes

Where applicable, are recommendations and next steps explained clearly for others to follow?

Partly

Is the rationale for the Open Letter provided in sufficient detail?

Yes

Reviewer Expertise:

Public health and demography

I confirm that I have read this submission and believe that I have an appropriate level of expertise to confirm that it is of an acceptable scientific standard.

# Reviewer report: Reviewer response for version 1

**DOI:** 10.21956/gatesopenres.14351.r29108

**Published:** 2020-09-02

**Authors:** Kenji Shibuya

**Affiliations:** 1 Institute for Population Health, King's College London, London, UK

This is a well written summary of key issues and future directions related to direct and indirect perinatal outcomes associated with COVID in low- and middle-income countries. The authors reviewed extensively the major maternal and perinatal risk factors of direct health outcomes from studies primarily done in high-income regions, but issues on indirect health outcomes, such as limited health service access (including other vaccines) and aggravating health inequality from lockdowns, and the impact of social determinants are somewhat limited. 

 The authors are advocating for the inclusion of pregnant women in the vaccine trials and the need for more basic research including immunology, but fail to address the lack of basic epidemiological data to track progress and monitor what works and what does not in low- and middle-income countries. This could be done in the context of ongoing efforts such as the Countdown and SDG monitoring processes.

 The future directions clearly depend on a global concerted effort on collecting, synthesizing and analyzing basic epidemiological data in low- and middle-income countries and the authors should address how this could be implemented and by whom.

Does the article adequately reference differing views and opinions?

Yes

Are all factual statements correct, and are statements and arguments made adequately supported by citations?

Partly

Is the Open Letter written in accessible language?

Yes

Where applicable, are recommendations and next steps explained clearly for others to follow?

Partly

Is the rationale for the Open Letter provided in sufficient detail?

Yes

Reviewer Expertise:

Health metrics and evaluation, health systems, global health policy.

I confirm that I have read this submission and believe that I have an appropriate level of expertise to confirm that it is of an acceptable scientific standard, however I have significant reservations, as outlined above.

# Response

**Published:** 2021-03-16

**Authors:** Claire von Mollendorf

**Affiliations:** Murdoch Childrens Research Institute, Australia

**Response to reviewer 1 comments:**

***We thank the reviewer for their comments and have responded to each of their points separately below*** ***:***

 The authors reviewed extensively the major maternal and perinatal risk factors of direct health outcomes from studies primarily done in high-income regions, but issues on indirect health outcomes, such as limited health service access (including other vaccines) and aggravating health inequality from lockdowns, and the impact of social determinants are somewhat limited.

***We have added some information into the review article which discusses the impact of pandemic on maternal and vaccination healthcare services:***

 “Early in the pandemic a study modelled the indirect effect of the pandemic on maternal and child health and resultant additional deaths in 118 LMICs using three possible scenarios (1). The scenarios varied in the extent of reduction in essential health intervention coverage (including a reduction in antenatal and postnatal care ranging from 18.5-51.9%) and increase in prevalence of wasting. It was estimated that under-5 child deaths could increase by 9.8–44.7% per month and maternal deaths by 8.3–38.6% per month depending on the associated disruptions (1). Data collected by the Population Council in five informal settlements in Nairobi in April 2020 found that women were twice as likely as men to miss essential health services. Missed services including family planning and antenatal care (2). In addition, midwives in Kenya, Uganda and Tanzania, reported a reduction in the number of women attending maternal health clinics, and an increase in delayed labour presentations to hospital and insufficient antenatal care (3).

 A systematic review which included LMICs evaluated the likely impact of the COVID-19 pandemic on immunisation coverage and the factors contributing to service disruption. The review found a reduction in vaccination coverage, reduction in doses administered and an increase in polio cases in polio endemic countries (4).”

 The authors are advocating for the inclusion of pregnant women in the vaccine trials and the need for more basic research including immunology, but fail to address the lack of basic epidemiological data to track progress and monitor what works and what does not in low- and middle-income countries. This could be done in the context of ongoing efforts such as the Countdown and SDG monitoring processes.

***We have added some information into the review article which discusses the roadmap for COVID-19 vaccines and the inclusion of pregnant women into vaccination programmes:***

 “Now that COVID-19 vaccines are available, the WHO SAGE committee has published a roadmap which outlines how to prioritise vaccination depending on vaccine availability and disease burden and risk. The roadmap acknowledges that pregnant women have been disadvantaged with regards to the development and use of vaccines in pandemics. Data needs to be generated for pregnant women and pregnant women should be included in roadmap stage 3 - when evidence should be available to determine whether the benefits of COVID-19 vaccination outweighs the risk of SARS-CoV2 infection and potential severe COVID-19 (5).”

 The future directions clearly depend on a global concerted effort on collecting, synthesizing and analyzing basic epidemiological data in low- and middle-income countries and the authors should address how this could be implemented and by whom.

***We have added some information into the review article which discusses collection of epidemiological data in LMICs:***

 “In terms of COVID-19 research in LMICs, it has been suggested that this is incorporated into public health and clinical activities to avoid these vital services being diverted, and also that this research should be driven by local investigators (6). The WHO has published a number of generic protocols for COVID-19 research and response, including surveillance, serology and case investigation (7). In 2020 the United Nations launched a global humanitarian response plan to fund the COVID-19 response in the poorest countries with both public and private sector support (8).”

 References:

 1.    Roberton T, Carter ED, Chou VB, Stegmuller AR, Jackson BD, Tam Y, et al. Early estimates of the indirect effects of the COVID-19 pandemic on maternal and child mortality in low-income and middle-income countries: a modelling study. Lancet Glob Health. 2020. 10.1016/S2214-109X(20)30229-1

 2.    Population Council. "Kenya: COVID-19 knowledge, attitudes and practices—Responses from Second Round of Data Collection in Five Informal Nairobi Settlements (Kibera, Huruma, Kariobangi, Dandora, Mathare)," COVID-19 Research & Evaluations Brief. Nairobi. 2020.

 3.    Pallangyo E, Nakate MG, Maina R, Fleming V. The impact of covid-19 on midwives' practice in Kenya, Uganda and Tanzania: A reflective account. Midwifery. 2020;89:102775. 10.1016/j.midw.2020.102775

 4.    Lassi ZS, Naseem R, Salam RA, Siddiqui F, Das JK. The Impact of the COVID-19 Pandemic on Immunization Campaigns and Programs: A Systematic Review. Int J Environ Res Public Health. 2021;18(3). 10.3390/ijerph18030988

 5.    World Health Organization. WHO SAGE Roadmap For Prioritizing Uses Of COVID-19 Vaccines In The Context Of Limited Supply. 2020.

 6.    Gupta M, Wahl B, Adhikari B, Bar-Zeev N, Bhandari S, Coria A, et al. The need for COVID-19 research in low- and middle-income countries. Global health research and policy. 2020;5:33. 10.1186/s41256-020-00159-y

 7.    World Health Organization. WHO’s country and technical guidance on COVID-19 2020 [Available from: https://www.who.int/emergencies/diseases/novel-coronavirus-2019/technical-guidance .

 8.    The United Nations Department of Global Communications. Funding the fight against COVID-19 in the world's poorest countries 2020. 2020 [Available from: https://www.un.org/en/un-coronavirus-communications-team/funding-fight-against-covid-19-world%E2%80%99s-poorest-countries .
